# Diagnostic delay in the early phase of psoriatic arthritis is independently associated with adverse pain-related cognitive responses: a multicentre cross-sectional study

**DOI:** 10.3389/fimmu.2026.1869035

**Published:** 2026-07-14

**Authors:** Eugenio Capparelli, Maria Iacovantuono, Marco Tasso, Sergio Del Vescovo, Benedetta Monosi, Paola Conigliaro, Paola Faggioli, Florenzo Iannone, Giuseppe Lopalco, Maria Sole Chimenti

**Affiliations:** 1 Department of Systems Medicine, Rheumatology, Allergology and Clinical Immunology, University of Rome Tor Vergata, Rome, Italy; 2 Rheumatology Unit, Azienda Socio Sanitaria Territorialie (ASST) Ovest Milanese, Legnano Hospital, Legnano, Italy; 3 Department of Clinical Medicine and Surgery, University of Naples Federico II, Naples, Italy; 4 Rheumatology Unit, Department of Precision and Regenerative Medicine and Ionian Area, University of Bari, Bari, Italy

**Keywords:** diagnostic delay, early recognition, Pain Catastrophizing Scale, pain-related cognitive burden, patient-reported outcomes, psoriatic arthritis

## Abstract

**Objective:**

Diagnostic delay during the early phase of psoriatic arthritis (PsA) is associated with worse long-term outcomes. Thus, early recognition remains crucial. Whether the delayed recognition of early PsA is associated with adverse pain-related cognitive responses has not been investigated. We examined the association between diagnostic delay, time to first biologic or targeted synthetic DMARD (b/tsDMARD) initiation, and pain-related cognitive burden in a multicentre cohort of patients with PsA.

**Methods:**

We conducted a multicentre cross-sectional study within three Italian referral centres for PsA. Adverse pain-related cognitive responses were investigated using the Pain Catastrophizing Scale (PCS), which captures the domains of rumination, magnification and helplessness. A total PCS score ≥ 30 was used as cut-off to define PCS-defined pain impairment. We defined: diagnostic delay as the lag time between symptoms onset and confirmed PsA diagnosis; therapeutic delay as the interval between PsA diagnosis and first b/tsDMARD initiation. Clinical, laboratory and patient-reported variables were compared according to PCS status. Multivariable logistic regression analysis was used to identify factors independently associated with pain catastrophizing.

**Results:**

Among 207 patients, 31.4% had PCS ≥30. Patients with PCS-defined impairment had significantly higher pain VAS, patient global assessment, evaluator global assessment, tender joint count, swollen joint count, ESR, DAPSA, BASDAI and ASDAS-CRP scores. Higher WPI, SSS and BDI scores were also reported amongst those with PCS ≥30. Diagnostic delay ≥6 months and therapeutic delay were observed to be more frequent in patients with PCS ≥30 (p=0.017 and p=0.045 respectively). Patients with delayed diagnosis had significantly higher PCS total score and within the subdomains of rumination, magnification and helplessness. The multivariable analysis showed the independent association of the diagnostic delay with PCS ≥30 (OR 3.65, 95% CI 1.21-11.02).

**Conclusion:**

Delayed recognition in the early phase of PsA is independently associated with adverse pain-related cognitive burden, as defined by the Pain Catastrophizing Scale.

## Introduction

Psoriatic arthritis (PsA) is a chronic inflammatory musculoskeletal disease characterised by marked clinical heterogeneity and substantial burden across pain, physical function and health-related quality of life (1). Although inflammation is central to disease pathogenesis, pain in PsA cannot be fully explained by inflammatory activity alone and may also be shaped by cognitive, affective and symptom-amplifying mechanisms (2, 3).

Previous evidence has demonstrated that higher diagnostic delay and time between symptom onset and the start of first-line therapy were observed in PsA compared to Rheumatoid Arthritis, where detectable disease-related autoantibodies serve as relevant markers to guide the diagnosis (4). Early recognition of PsA remains therefore a major unmet clinical need, as diagnostic delay during the early phase of disease has been associated with worse clinical outcomes, poorer physical function and progression towards structural damage (5–7). Conceptually, the longer the diagnostic delay, the greater the likelihood of adverse outcomes is. Pointing it out, Zundell MP et al., in a large cohort of PsA patients, assessed diagnostic delay at different time points (7–12 months, 12–24 months or greater than 24 months), finding out that even a diagnostic delay greater than six months should be considered unacceptable as almost one-third of cases reported worse outcomes on depression and social ability sphere.

Accordingly, increasing attention has been directed towards identifying the clinical consequences of missed opportunities for recognition during the early phase of PsA (4).

Among the non-inflammatory contributors to symptom burden, pain-related cognitive responses are increasingly recognised as clinically relevant (8–10). In the present study, this dimension was operationalised using the Pain Catastrophizing Scale (PCS), a validated questionnaire that captures three core domains of maladaptive pain-related cognition: rumination, magnification and helplessness (11). While the PCS has traditionally been used to assess pain catastrophizing, the broader framing of adverse pain-related cognitive responses may better reflect the multidimensional clinical profile captured in patients with inflammatory arthritis (12).

Previous studies in PsA have shown that higher PCS scores are associated with greater disease activity, difficult-to-treat status, metabolic comorbidity and reduced biologic retention (13–16). However, whether delayed recognition and missed therapeutic opportunities during the early phase of PsA are associated with increased PCS scores has not been specifically investigated.

We therefore aimed to assess the relationship between diagnostic delay, time to first biologic or targeted synthetic DMARD (b/tsDMARD) initiation, and adverse pain-related cognitive responses in a multicentre cohort of patients with PsA.

## Methods

### Study design and setting

We conducted a multicentre cross-sectional study across three Italian tertiary referral rheumatology centres with dedicated outpatient services for PsA: “Tor Vergata University” in Rome, the “University Hospital Consortium Polyclinic” of Bari and the “University of Naples Federico II”. Consecutive patients were recruited during routine clinical visits. The study was designed to investigate whether delayed recognition and delayed treatment during the early phase of PsA were associated with adverse pain-related cognitive responses assessed at the time of study evaluation.

Although patients were evaluated cross-sectionally during established disease, diagnostic and treatment timelines were retrospectively reconstructed to capture the timing of first symptoms onset, diagnosis and treatment initiation in the early phase of PsA.

### Study population

Adult patients (≥ 18 years old), with a confirmed diagnosis of PsA, fulfilling the 2006 ClASsification for Psoriatic ARthritis (CASPAR) criteria (17), were eligible for inclusion and were consecutively enrolled.

Patients were included if they were able to complete the study questionnaires and if sufficient clinical information were available to reconstruct the timeline of disease onset and treatment initiation. Patients with incomplete data for the main timing variables or PCS assessment were excluded from the analysis.

### Data collection

Demographic and clinical data were obtained from medical records and structured clinical assessment at the study visit. The following variables were recorded: sex, age, body mass index (BMI), smoking status, alcohol consumption, age at symptom onset, age at PsA diagnosis, disease duration, PsA clinical domains, comorbidities and treatment history.

Disease domains included cutaneous psoriasis, nail psoriasis, enthesitis, dactylitis, axial involvement, non-infectious uveitis and inflammatory bowel disease. Overall comorbidity burden was also assessed, and the Charlson Comorbidity Index (CCI) was calculated.

### Definition of diagnostic and therapeutic timelines

Diagnostic delay was defined as the time interval between patient-reported onset of musculoskeletal symptoms attributable to PsA with either inflammatory or non-inflammatory characteristics and confirmed PsA diagnosis. To reflect timeliness of recognition during the early phase of disease, diagnostic delay was analysed both as a continuous variable, expressed in months, and as a categorical variable using a prespecified threshold of <6 months versus ≥6 months.

Therapeutic delay was defined as the time interval between confirmed PsA diagnosis and initiation of the first b/tsDMARD. This variable was analysed both continuously, expressed in years, and categorically as <1 year versus ≥1 year from diagnosis. We intentionally missed to consider therapeutic delay as the time interval between diagnosis (or symptoms onset) and conventional synthetic DMARDs initiation as most of recruited patients underwent csDMARDs prescription alongside the diagnosis of PsA. This could have determined a non-elidable grade of overlap between diagnostic and therapeutic delay.

### Assessment of adverse pain-related cognitive responses

Adverse pain-related cognitive responses were assessed using the Pain Catastrophizing Scale (PCS), a validated self-administered questionnaire evaluating three core domains of maladaptive pain-related cognition: rumination, magnification and helplessness (18). Total PCS score and subdomain scores were recorded for all participants.

For the main analysis, a total PCS score ≥30 was used to identify clinically relevant adverse pain-related cognitive burden. Despite a total PCS score of 30 has not yet been validated in specific PsA populations, it was employed as it corresponds to the 75th percentile of the distribution of PCS scores in clinic samples of chronic pain patients (19).

In addition to the dichotomous PCS classification, PCS total score and PCS subdomain scores were analysed as continuous variables in relation to diagnostic and therapeutic delay.

### Clinical assessment and patient-reported outcomes

At the study visit, the following disease activity and symptom-related measures were collected: pain visual analogue scale (VAS), patient global assessment (PGA), evaluator global assessment (EGA), tender joint count (TJC, 68 joints), swollen joint count (SJC, 66 joints), Leeds Enthesitis Index (LEI), erythrocyte sedimentation rate (ESR) and C-reactive protein (CRP).

Composite disease activity indices included the Disease Activity index for Psoriatic Arthritis (DAPSA). The Bath Axial Sponsyloarthrtis Disease Activity Index (BASDAI) and the Axial Sponsyloarthrtis Disease Activity Score using CRP (ASDAS-CRP) were also recorded in patients with axial involvement.

To better characterise the broader symptom-related and cognitive-affective profile associated with PCS-defined impairment, additional patient-reported and symptom-related measures were collected, including the Beck Depression Inventory (BDI), Widespread Pain Index (WPI) and Symptom Severity Scale (SSS) (20, 21).

### Outcome measures

The primary outcome was the presence of clinically relevant adverse pain-related cognitive burden, defined as PCS ≥30.

Secondary analyses explored: the association between diagnostic delay and PCS total and subdomain scores; the association between therapeutic delay and PCS total and subdomain scores; differences in disease activity, symptom burden and patient-reported outcomes according to PCS status.

### Statistical analysis

Continuous variables are reported as mean ± standard deviation (SD) or median and interquartile range (IQR), according to variable distribution. Categorical variables are reported as number and percentage.

Comparisons between patients with PCS <30 and PCS ≥30 were performed using Student’s *t*-test for normally distributed continuous variables or the Mann-Whitney *U* test for non-normally distributed variables, as appropriate. Categorical variables were compared using the chi-square test or Fisher’s exact test.

Associations between delayed diagnosis, delayed treatment initiation and PCS total or subdomain scores were explored in univariate analyses.

A multivariable binary logistic regression model was constructed to identify factors independently associated with PCS ≥30. The outcome variable was PCS ≥30. Based on clinical relevance, the following covariates were included in the model: sex, BMI, age at PsA diagnosis, diagnostic delay ≥6 months, therapeutic delay ≥1 year and BDI. Potential confounders which could have produced collider bias and overadjustment have been intentionally excluded from the model and encompass the presence of fibromyalgia as assessed by WPI and SSS, pain-VAS, DAPSA and CCI. Results are presented as odds ratios (ORs) with 95% confidence intervals (CIs). A two-sided *p* value <0.05 was considered statistically significant.

### Ethics

The study was conducted in accordance with the principles of the Declaration of Helsinki and local institutional requirements. All participants provided informed consent prior to enrolment. Approval from the relevant ethics committees or institutional review boards was obtained according to local regulations. Protocol number of Fondazione Policlinico Tor Vergata University was 186/16.

## Results

### Patient characteristics

A total of 207 patients were included in the study. Of these, 65 (31.4%) had PCS ≥30 and were classified as having clinically relevant adverse pain-related cognitive burden, whereas 142 (68.6%) had PCS <30.

The two groups were broadly comparable in terms of sex distribution, BMI, age at enrolment, age at symptom onset, age at diagnosis and disease duration (**Table 1**). Diagnostic delay, considered as a continuous variable, was numerically longer amongst patients with PCS ≥30, although the difference did not reach statistical significance. However, the proportion of patients with diagnostic delay ≥6 months was significantly higher in the PCS ≥30 group than in the PCS <30 group (89.2% vs 74.6%, p=0.017).

**Table 1:** Patient’s main characteristics.

Patients features	Total Cohort N = 207	PCS <30 N = 142	PCS ≥30 N = 65	p-value
**Female sex, *n (%)***	129 (62.3)	89 (62.7)	40 (61.5)	0.878
**Body Mass Index (Kg/m** ^2^ **), *mean ± S.D.***	25.8 ± 4.4	25.6 ± 4.1	26.3 ± 5.0	0.314
**Underweight, *n (%)***	2 (1)	1 (0.7)	1 (1.5)	0.531
**Normal weight, *n (%)***	102 (49.3)	71 (50)	31 (47.7)	0.767
**Overweight, *n (%)***	69 (33.3)	48 (33.8)	21 (32.3)	0.875
**Obese, *n (%)***	34 (16.4)	22 (15.5)	12 (18.5)	0.686
**Age at enrolment, years, *mean ± S.D.***	55.3 ± 11.2	55.5 ± 11.6	54.8 ± 10.7	0.701
**Age at symptoms onset, years, *mean ± S.D.***	41.7 ± 12.2	42.5 ± 12.4	40.1 ± 11.6	0.178
**Age at diagnosis, years, *mean ± S.D.***	45.1 ± 12.5	45.9 ± 12.4	44.2 ± 11.6	0.358
**Disease Duration, years, *median (IQR_1-3_)***	9 (4-16)	9 (4-16)	9 (5-14)	0.270
**Diagnostic Delay, months, *median (IQR_1-3_)***	24 (12-56)	20 (6-48)	24 (12-67)	0.134
**Diagnostic Delay ≥ 6 months, *n (%)*** **Diagnostic Delay < 6 months, *n (%)***	164 (79.2) 43 (20.8)	106 (74.6) 36 (25.4)	58 (89.2) 7 (10.8)	**0.017**
**Therapeutic delay, years, *median (IQR_1-3_)*** **b/tsDMARDs initiation ≥ 1 year from diagnosis, *n (%)*** **b/tsDMARDs initiation < 1 year from diagnosis, *n (%)***	4 (2-10) 168 (81.2) 39 (18.8)	4 (2-10) 111 (78.2) 31 (21.8)	6 (2-13.5) 57 (87.7) 8 (12.3)	**0.045** 0.127
**Smoking habit, *n (%)***	52 (25.1)	36 (25.4)	16 (24.6)	1.0
**Alcohol Consumption, *n (%)***	22 (10.6)	17 (12)	5 (7.7)	0.469
DISEASE DOMAINS, n (%)				
**Cutaneous Psoriasis**	152 (73.4)	111 (78.2)	41 (63.1)	**0.028**
**Enthesitis**	127 (61.4)	79 (55.6)	48 (73.8)	**0.014**
**Dactylitys**	44 (21.3)	32 (22.5)	12 (18.5)	0.585
**Nail Psoriasis**	57 (27.5)	39 (27.5)	18 (27.7)	1.0
**Axial involvement**	74 (35.7)	47 (33.1)	27 (41.5)	0.275
**NIU**	8 (3.9)	6 (4.2)	2 (3.1)	1.0
**Inflammatory Bowel Disease**	16 (7.7)	9 (6.3)	7 (10.8)	0.337
COMORBIDITIES, n (%)				
**Cardiovascular diseases**	80 (38.6)	53 (37.3)	27 (41.5)	0.645
**Type 2 Diabetes**	28 (13.5)	14 (9.9)	14 (21.5)	**0.029**
**Dyslipidemia**	74 (35.7)	47 (33.1)	27 (41.5)	0.275
**Hyperuricemia**	33 (15.9)	17 (12)	16 (24.6)	**0.025**
**Osteoporosis**	37 (17.9)	23 (16.2)	14 (21.5)	0.434
**Asthma/COPD**	25 (12.1)	17 (12)	8 (12.3)	1.0
**Previous Malignancies**	16 (7.7)	6 (4.2)	10 (15.4)	**0.010**
**Anxiety/depression**	24 (11.6)	13 (9.2)	11 (16.9)	0.159

%, percentage; COPD, chronic obstructive pulmonary disease, IQR, Interquartile range; NIU, noninfectious uveitis; n, number; TAS, Toronto Alexithymia Scale.

Bold values highlight significant p-values.

Median time to first b/tsDMARD initiation was significantly longer in patients with PCS ≥30 than in those with PCS <30 (6 years [IQR 2-13.5] vs 4 years [IQR 2-10], p=0.045). By contrast, the proportion of patients initiating b/tsDMARDs ≥1 year after diagnosis did not significantly differ between groups.

Among disease domains, patients with PCS ≥30 had a higher prevalence of enthesitis, whereas cutaneous psoriasis was more frequent in patients with PCS <30. With regard to comorbidities, type 2 diabetes, hyperuricaemia and previous malignancies were more prevalent in patients with PCS ≥30.

### Disease activity, symptom burden and patient-reported outcomes

Patients with PCS ≥30 had significantly higher pain VAS, PGA, EGA and TJC than those with PCS <30 (all p<0.001) (**Table 2**). SJC and ESR were also higher in the PCS ≥30 group, although objective inflammatory measures showed less consistent differences overall. No significant difference was observed in CRP, and LEI showed only a borderline difference between groups.

**Table 2:** Disease activity scores and patient reported outcomes at last follow-up according to PCS ≥30.

Outcome measures	PCS < 30	PCS ≥ 30	p-value
**Pain VAS (0-10), *mean ± S.D.***	3.5 ± 2.4	6.2 ± 3.0	**<0.001**
**PGA (0-10), *mean ± S.D.***	3.2 ± 2.2	5.2 ± 2.4	**<0.001**
**EGA (0-100), *median (IQR_1-3_)***	20 (1.5-40)	40 (27.5-70)	**<0.001**
**TJC (0-68), *mean ± S.D.***	1.6 ± 2.5	4.1 ± 4.3	**<0.001**
**SJC (0-66), *mean ± S.D.***	0.3 ± 0.8	1.2 ± 2.8	**0.015**
**LEI (0-6), *mean ± S.D.***	0.4 ± 1.2	0.8 ± 1.7	0.057
**DAPSA, *mean ± S.D.***	9.5 ± 6.5	13.9 ± 8.1	**<0.001**
**BASDAI (for axial inv.), *mean ± S.D.***	2.8 ± 1.8	5.2 ± 2.5	**<0.001**
**ASDAS-CRP (for axial inv.), *mean ± S.D.***	2.2 ± 1.6	3.6 ± 1.5	**<0.001**
**CRP (0-5) mg/L, *median (IQR_1-3_)***	0.45 (0.11–0.91)	0.33 (0.17-0.68)	0.819
**ESR (0-20) mm/h, *median (IQR_1-3_)***	10 (5- 15)	10 (7-24)	**0.040**
**WPI+SSS, *mean ± S.D.***	6.6 ± 5.4	13.5 ± 5.8	**<0.001**
**WPI, *mean ± S.D.***	3.3 ± 2.8	6.6 ± 3.3	**<0.001**
**SSS, *mean ± S.D.***	3.5 ± 3.1	6.9 ± 3.4	**<0.001**
**BDI, *median (IQR_1-3_)***	6.9 ± 7.7	13.3 ± 8.1	**<0.001**

ASDAS-CRP, Axial Spondyloarthritis Disease Activity Score–C-Reactive Protein; BASDAI, Bath Axial Spondyloarthritis Disease Activity Index; BDI, Beck Depression Inventory; CRP, C-Reactive Protein; DAPSA-CRP, Disease Activity index for Psoriatic Arthritis including C-Reactive Protein; EGA, Evaluator Global Assessment; ESR, Erythrocyte Sedimentation Rate; LEI, Leeds Enthesitis Index; PCS, Pain Catastrophizing Scale; PGA, Patient Global Assessment; SJC, Swollen Joint Count; SSS, Symptom Severity Scale; TAS, Toronto Alexithymia Scale; TJC, Tender Joint Count; VAS, Visual Analogue Scale; WPI, Widespread Pain Index.

Bold values highlight significant p-values.

Composite disease activity indices, including DAPSA, BASDAI and ASDAS-CRP, were all significantly higher in patients with PCS ≥30. In addition, symptom-related and psychological measures were markedly worse in this group, including WPI, SSS and BDI (all p<0.001).

### PCS-defined burden according to diagnostic and therapeutic delay

**Figure 1:**
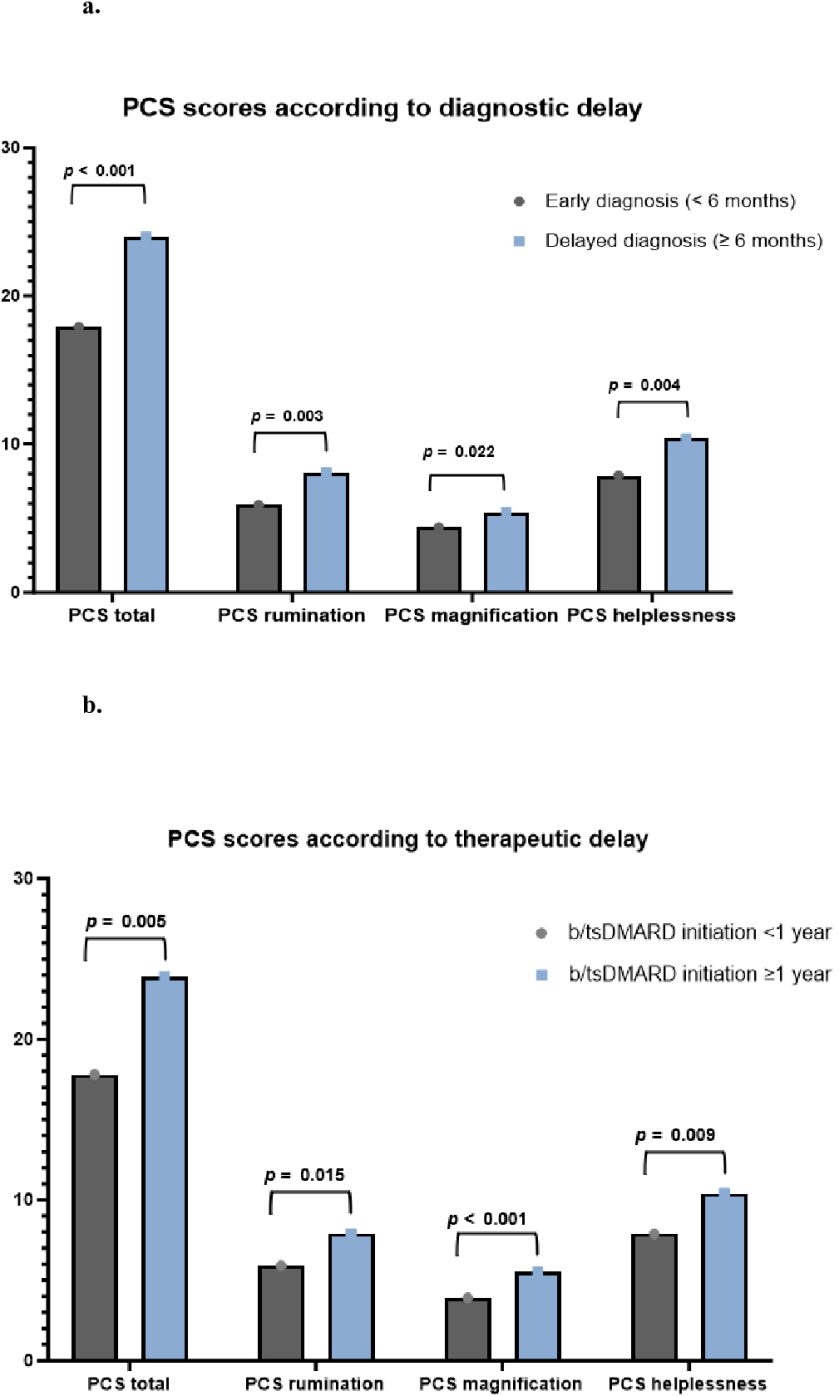
Bar chart showing PCS total score and main domains values by comparing PsA patients according to diagnostic delay with a threshold of 6 months **(a)**; and therapeutic delay with a threshold of 1 year **(b)**.

Patients with diagnostic delay ≥6 months had significantly higher PCS total scores than those diagnosed within 6 months (24.0 ± 11.9 vs 17.9 ± 9.8) (**Figure 1a**). They also had higher scores in each PCS subdomain, including rumination (8.1 ± 4.6 vs 5.9 ± 3.9), magnification (5.4 ± 2.3 vs 4.4 ± 2.4) and helplessness (10.4 ± 5.9 vs 7.9 ± 4.6).

Similarly, patients with delayed initiation of b/tsDMARDs had higher PCS total and subdomain scores in unadjusted analyses, including total PCS (23.9 ± 11.5 vs 17.8 ± 11.7), rumination (7.9 ± 4.4 vs 5.9 ± 4.7), magnification (5.5 ± 2.2 vs 3.9 ± 2.5) and helplessness (10.4 ± 5.7 vs 7.9 ± 5.2) (**Figure 1b**).

### Multivariable analysis

In multivariable logistic regression analysis, adjusted for confounders, the diagnostic delay ≥6 months was independently associated with PCS ≥30 (OR 3.65, 95% CI 1.21-11.02, p=0.022), along with BDI (OR 1.13, 95% CI 1.08-1.18, p<0.001). Age at PsA diagnosis, conversely, exerted a protective effect on PCS (OR 0.96, 95% CI 0.93-0.99, p=0.006) No significant independent association was found for therapeutic delay ≥1 year (**Table 3**).

**Table 3:** Binary logistic regression assessing predictors of PCS ≥ 30 (dependent variable).

PCS ≥30 (dependent variable)	Odds ratio	95% Confidence interval	Significance
Sex (male as reference)	0.68	0.34-1.39	0.290
BMI	1.04	0.96-1.11	0.324
Age at diagnosis	**0.96**	**0.93-0.99**	**0.006**
Diagnostic Delay ≥ 6 months	**3.65**	**1.21-11.02**	**0.022**
Therapeutic delay ≥ 1 year	1.19	0.43-3.32	0.742
BDI	**1.13**	**1.08-1.18**	**<0.001**

BMI, Body mass index; PCS, Pain Catastrophizing Scale; BDI, Beck’s Depression Inventory.

Bold values highlight significant p-values.

## Discussion

In this multicentre cross-sectional study, PCS was common in PsA as up to one third of our population reported a score ≥30. These patients exhibited a substantially greater burden across pain intensity, global disease assessment, depressive symptoms, widespread pain-related complaints and symptom severity. Taken together, these findings support the interpretation of pain catastrophizing as part of a broader symptoms-related and cognitive-affective trait, rather than as a simple proxy for objective inflammatory activity alone (22).

The main finding was that diagnostic delay ≥6 months, interpreted here as delayed recognition during the early phase of PsA, was independently associated with PCS whereas therapeutic delay did not retain significance after multivariable adjustment. These findings indicate that delayed recognition during the early phase of PsA, rather than delayed initiation of advanced targeted therapy, represents a critical aspect of PsA clinical spectrum.

A central implication of our findings concerns the critical importance of recognizing PsA in its early phases. PsA, compared to other rheumatic disease like RA, was found to be concerned by increased diagnostic and therapeutic delays due to several factors (23). The most relevant are represented by the absence of disease related serological markers and specific inflammatory indices as, for instance, ESR and CRP can be within the normal range at onset (24). Second, the musculoskeletal symptoms, such as joint and entheseal pain, can be insidious at onset and have been reported with inflammatory and non-inflammatory characteristics. This is particularly relevant in patients with obesity and at younger age, where the demand of mechanical load is usually heightened. This can lead to misinterpretation and underestimation also by physicians who cannot clearly discriminate the nature of pain (25, 26). Third, the absence of cutaneous psoriasis in almost 15% of PsA cases and the presence of further extra-musculoskeletal manifestations, which parallels the multidomain nature seen in established disease, could complicate the diagnosis and the initiation of a proper treatment (27, 28). Lastly, our previous evidence demonstrated that neither the presence of cutaneous psoriasis was associated with early diagnosis (4). This evidence was further supported by a larger prospective study, where up to a half of newly diagnosed Psoriatic patients reported inflammatory and non-inflammatory musculo-skeletal symptoms at baseline, however without developing PsA in a two-year follow-up (29).

However, since early recognition of PsA can halt structural damage and avoid worse functional outcomes, it seems hard to obtain and therefore given these premises we considered diagnostic delay as a clinically meaningful exposure variable (5). In accordance, our data suggest that delayed recognition may extend beyond inflammatory and structural domains, as patients experiencing a longer interval between symptom onset and diagnosis had higher PCS total scores and higher scores in the PCS subdomains of rumination, magnification and helplessness.

Although the PCS is conventionally used to assess pain catastrophizing, our findings suggest that the observed profile cannot be reduced to pain intensity alone. It instead reflects a broader cognitive-affective burden, as suggested by the significant association between PCS and higher BDI in multivariable model. This interpretation is also supported by the dissociation between PCS and objective inflammatory measures. While patients with PCS ≥30 had higher ESR and differences in composite disease activity indices, CRP and LEI did not differ between groups. PCS, indeed, is not simply a surrogate of inflammatory activity but it may identify a subgroup of patients exhibiting a strict interaction between inflammation, negative emotional pain appraisal, and depressive symptoms (13).

In this regard, prolonged and uncontrolled exposure to inflammatory stimuli might be linked to altered pain processing, further favouring central sensitization and altered cognitive-emotional processing of pain itself. This was also confirmed by effect of BDI on PCS in our population, further supporting the co-existence of an intricate relationship between inflammation, central sensitization and altered cognitive-affective responses. Timely diagnosis may influence not only treatment decisions and structural outcomes, but also the longer-term cognitive-affective framing of pain. From a translational perspective, these findings reinforce the concept that the early phase of PsA represents a critical window not only for inflammatory control, but also for reducing the likelihood of adverse pain-related cognitive patterns.

Our previous evidence confirmed the association between diagnostic delay and the presence of further maladaptive cognitive issues like kinesiophobia. Kinesiophobia, being emotionally and cognitively determined, refers to an avoidant behavioural strategy, where affected patients tend to reduce movement and physical activity not only to diminish current pain intensity but also to condense worries about future pain sensations. Thus, increasing prolonged physical inactivity and facilitating the emergence of disability and mood disorders, like depression and anxiety (30).

By contrast, therapeutic delay showed no association with PCS. Although longer time to first b/tsDMARD initiation was associated with higher PCS scores in unadjusted analyses, this variable was not independently associated with PCS ≥30 after adjustment. This finding should be interpreted cautiously, since time to first b/tsDMARD initiation may not fully reflect therapeutic delay in a strict sense, being influenced by disease phenotype, historical prescribing patterns, prior conventional treatment exposure and local treatment strategies (31).

Several limitations should be acknowledged. First, the cross-sectional design precludes causal inference, and the temporal relationship between delayed recognition of early PsA and adverse pain-related cognitive responses cannot be definitively established. Specifically, data regarding the presence of an “adverse cognitive responses tendency” to pain prior the diagnosis of PsA are lacking in our population, making it difficult to assess their potential influence on patients’ symptoms reporting at onset, which could have further impacted the promptness of the diagnosis. Therefore, the coexistence of reverse causality between the association of diagnostic delay and maladaptive cognitive responses cannot be definitively excluded from the present study and should be acknowledged as a major limitation. Secondly, diagnostic delay was reconstructed retrospectively and depends in part on patient-reported timing of symptom onset, making recall bias possible. Thirdly, the definition of therapeutic delay based on the time from diagnosis to first b/tsDMARDs initiation represents an important limitation. Indeed, this interval may be influenced by several factors, such as disease domains distribution, previous csDMARDs exposure, historical treatment paradigms, reimbursement and access policies. However, the rationale of defining therapeutic delay with the b/tsDMARDs initiation date was supported by the evidence that most of recruited patients underwent csDMARDs alongside PsA diagnosis, making it difficult to interpret therapeutic delay distinct from diagnostic delay. Consequently, a sort of overlapping between the two intervals should be considered for cohorts coming from tertiary care settings. Moreover, the limited restrictions on advanced multi-domain targeting therapy access in referral centres made it suitable to use a cut-off of one year. Moreover, residual confounding cannot be excluded, particularly for variables that may lie along the pathway linking disease burden and maladaptive pain-related cognition, such as fibromyalgia. Finally, although the early phase of PsA is central to the interpretation of our findings, the present cohort consisted of patients assessed during established disease rather than an inception cohort of early PsA.

Despite these limitations, the multicentre design and the integration of clinical, laboratory and patient-reported measures provide a clinically meaningful perspective on the longer-term consequences of delayed recognition during the early phase of PsA.

## Conclusions

Overall, our findings support the evidence that delayed recognition of PsA during its early phases could exert a negative impact not only on inflammatory outcomes but also long-term symptom interpretation and cognitive-affective disease burden. Thus, screening and directing clinical attention towards adverse cognitive outcomes should be practiced especially in patients reporting diagnostic delay.

## Data Availability

Additional files can be requested to the corresponding author who will review the request and accomplish to the diffusion of data case by case. Requests to access the datasets should be directed to maria.sole.chimenti@uniroma2.it.
